# Potassium availability triggers *Mycobacterium tuberculosis* transition to, and resuscitation from, non-culturable (dormant) states

**DOI:** 10.1098/rsob.140106

**Published:** 2014-10-15

**Authors:** Elena G. Salina, Simon J. Waddell, Nadine Hoffmann, Ida Rosenkrands, Philip D. Butcher, Arseny S. Kaprelyants

**Affiliations:** 1 Institution of the Russian Academy of Sciences A.N. Bach Institute of Biochemistry RAS, Moscow, Russia; 2 Brighton and Sussex Medical School, University of Sussex, Brighton, UK; 3 Department of Infectious Disease Immunology, Statens Serum Institut, Copenhagen, Denmark; 4 Institute for Infection and Immunity, St George's University of London, London, UK

**Keywords:** tuberculosis, dormancy, latency, transcriptional profiling, resuscitation, potassium

## Abstract

Dormancy in non-sporulating bacteria is an interesting and underexplored phenomenon with significant medical implications. In particular, latent tuberculosis may result from the maintenance of *Mycobacterium tuberculosis* bacilli in non-replicating states in infected individuals. Uniquely, growth of *M. tuberculosis* in aerobic conditions in potassium-deficient media resulted in the generation of bacilli that were non-culturable (NC) on solid media but detectable in liquid media. These bacilli were morphologically distinct and tolerant to cell-wall-targeting antimicrobials. Bacterial counts on solid media quickly recovered after washing and incubating bacilli in fresh resuscitation media containing potassium. This resuscitation of growth occurred too quickly to be attributed to *M. tuberculosis* replication. Transcriptomic and proteomic profiling through adaptation to, and resuscitation from, this NC state revealed a switch to anaerobic respiration and a shift to lipid and amino acid metabolism. High concordance with mRNA signatures derived from *M. tuberculosis* infection models suggests that analogous NC mycobacterial phenotypes may exist during disease and may represent unrecognized populations *in vivo*. Resuscitation of NC bacilli in potassium-sufficient media was characterized by time-dependent activation of metabolic pathways in a programmed series of processes that probably transit bacilli through challenging microenvironments during infection.

## 2. Introduction

More than 1.5 million deaths occur every year as a result of *Mycobacterium tuberculosis* infection. Latent infection is widely distributed, with one-third of the world population estimated to be infected with *M. tuberculosis* without overt symptoms of disease. Infected individuals have a lifelong risk of disease reactivation of approximately 5%, which increases to an estimated 10% per annum in immuno-compromised patients [1]. The location and physiological state of bacteria in latent disease is poorly understood, which has hindered efforts to develop novel strategies to combat tuberculosis reactivation.

The clinical state of latent *M. tuberculosis* infection is traditionally associated with the transition of bacilli to a dormant state in response to non-optimal growth conditions *in vivo* resulting from activation of the host immune system. However, the mechanisms mediating *M. tuberculosis* dormancy and subsequent reactivation are unclear. Dormancy is a specific physiological state accompanied by significant cessation of metabolic activity. Many studies have modelled this physiological state both *in vivo* [2–6] and *in vitro* [7–14]. Dormant bacilli isolated from the majority of these *in vitro* models are fully culturable, whereas bacilli extracted from *in vivo* models of latent disease are characterized by non-culturability [15,16]. Here, we use non-culturability as an operational term to describe the inability of cells to produce colonies on non-selective solid media under defined conditions. We define the transition of *M. tuberculosis* between culturable and non-culturable (NC) states and through the resuscitation of mycobacterial growth, reflecting the reactivation of latent tuberculosis disease.

*Mycobacterium tuberculosis* bacilli are exposed to multiple microenvironments during infection, from dynamic intracellular compartments to heterogeneous lung lesion architectures to an extracellular milieu in lesions and in sputum. Therefore, in addition to the antimicrobial defence mechanisms that bacilli encounter during infection, bacilli must also adapt to wide changes in ion concentrations associated with both intracellular and extracellular lifestyles. Recent studies have highlighted the importance of mycobacterial strategies to control both zinc [17] and copper [18,19] levels. Potassium (K^+^) is crucial for maintaining an electrochemical gradient and a proton-motive force, as well as regulating intracellular pH and osmotic pressure in both eukaryotic and mycobacterial cells [20,21]. K^+^ is concentrated inside both bacterial and eukaryotic cells [22], with concentrations ranging from 0.1 to 1 M in bacteria [23] and approximately 140 mM in eukaryotic cells, while K^+^ levels in surrounding fluids are at least 20 times lower [24]. Low K^+^ concentrations may result in the inability of bacilli to maintain pH_in_ values at acceptable levels in mildly acidic conditions [25], which has been demonstrated to be highly deleterious for *M. tuberculosis* viability [26].

*Mycobacterium tuberculosis* has several tightly regulated strategies to sense and adapt to these extreme changes in potassium gradient. The regulation of potassium transport is controlled by the Trk and Kdp systems in mycobacteria. The major constitutive *M. tuberculosis* potassium transporter consists of two Trk proteins, CeoB and CeoC. The Kdp two-component system is inducible and encoded by *kdpFABCDE* [22,24]. The imbalance of K^+^ transport that resulted from disruption of *trkA* in *Mycobacterium smegmatis* led to severe defects in membrane potential and intracellular pH especially in acid media [21]. Similarly, inhibition of potassium transport led to the build up of H^+^ resulting in the destruction of *M. tuberculosis* bacilli in macrophages [27]. These findings are supported by whole-genome essentiality studies that identify the kdp system (*kdpE*) as required for *in vitro* growth [28] and both *kdpE* and *ceoB* as necessary for successful growth in macrophages [29]. Furthermore, by using the transcriptional induction of *kdp* or *ceo* genes as a bioprobe for low potassium levels, multiple potassium-limited *in vivo* environments may be recognized, including a murine hollow fibre model of extracellular bacilli [6], the human lung [30] and human-derived sputa [31]. We therefore reasoned that changes in K^+^ concentration may induce bacterial responses leading to dormancy that enables bacilli to survive long-term *in vivo*. We recently reported the presence of *M. tuberculosis* bacilli in an NC state after limitation of potassium [32]. In this study, extending our previous findings, we determine that potassium limitation is an environmental cue for the formation of NC *M. tuberculosis* bacilli in nutrient-sufficient aerobic conditions; we define this physiological state using a combination of transcriptome and proteome approaches; and we characterize *M. tuberculosis* recovery and re-growth when potassium is re-introduced, modelling the reactivation of latent disease.

## 3. Material and methods

### 3.1. Organism and media

*Mycobacterium tuberculosis* strain H37Rv was initially grown for 10 days in Sauton medium, containing: KH_2_PO_4_, 0.5 g; MgSO_4_ · 7H_2_O, 1.4 g; l-asparagine, 4 g; glycerol, 60 ml; ferric ammonium citrate, 0.05 g; sodium citrate, 2 g; 1% ZnSO_4_ · 7H_2_O, 0.1 ml; H_2_O, to 1 l; pH 7.0 (adjusted with 1 M NaOH) supplemented with ADC [33] and Tween-80 (0.05% v/v) at 37°C and shaken at 200 r.p.m. For a high most probable number (MPN)/low colony forming units (CFU) model of NC bacilli, starter cultures were then sub-cultured into fresh media (same composition) and incubated for another 10 days (OD_600_ 3.5–4.0) before inoculating into either fresh complete Sauton or potassium-deficient Sauton media, where potassium ions were eqiumolarly substituted by sodium ions, both supplemented with ADC and Tween-80. Cultures were incubated in K^+^-deficient Sauton media at 37°C and shaken at 200 r.p.m. for 39–41 days in loose-capped flasks. For a high MPN/high CFU second model variant of NC bacilli, starter cultures were sub-cultured into fresh media and then incubated for another 15 days before inoculating into potassium-deficient Sauton media.

### 3.2. Estimation of viability

Ten-fold serial dilutions of *M. tuberculosis* cultures were prepared and plated in triplicate onto solidified Sauton medium agar. Plates were incubated at 37°C for 25 days followed by enumeration of CFU. The same dilutions in triplicate were also used to assay MPN in 48-well plates, providing an estimation of the proportion of bacteria able to grow on solid and in liquid media, respectively. The 48-well plates were incubated statically at 37°C for 30 days. Wells with visible bacterial growth were counted as positive, and MPN values were calculated using standard statistical methods [34].

### 3.3. Resuscitation of non-culturable cells

For both high MPN/low CFU and high MPN/high CFU model variants, NC cells were harvested by centrifugation (20 min at 5000 r.p.m.), washed twice with fresh Sauton media and resuspended in ‘resuscitation media’ which is standard Sauton medium containing 0.6% glycerol and supplemented with an equal volume of used culture supernatant, prepared as previously described [35], and Tween-80 (0.05% v/v). In contrast to our previous model [32], the addition of culture supernatant was required for maximum cell recovery from undiluted dense cultures that were needed for proteomics and transcriptomics platforms. Bacterial cultures were incubated with agitation at 37°C and harvested at appropriate time points for CFU counting, radioactive uracil incorporation, and isolation of RNA and protein.

### 3.4. Incorporation of radioactively labelled uracil

One microlitre of 5,6,-^3^H uracil (1 µCi) was added to 1 ml culture samples and incubated at 37°C with agitation for 20 h. Two hundred microlitres of this culture was placed in 3 ml 7% ice-cold CCl_3_COOH and incubated at 0°C for 20 min, followed by filtration through a glass microfibre filter (Whatman). Precipitated cells were washed with 3 ml 7% CCl_3_COOH and 6 ml 96% ethanol. Filters were placed in 10 ml scintillation mixture; impulse counts were determined by LS analyser (Beckman Instruments) and expressed as counts per minute (cpm).

### 3.5. Antibiotic sensitivity

Logarithmic phase cells grown in standard Sauton media and NC cells from potassium-deficient Sauton media were exposed to rifampicin (5 µg ml^−1^), isoniazid (1 µg ml^−1^) and benzothiazinone-043 (0.2 µg ml^−1^) [36] for 7 days at 37°C. The bacilli were washed and resuspended in fresh culture media as for the resuscitation of NC cells. The degree of killing was then determined in liquid medium by MPN assay.

### 3.6. Transmission electron microscopy

Bacilli were pelleted by centrifugation and fixed, followed by additional fixing with 1% solution of osmium tetroxide in 0.05 M cacodylate buffer (pH 7.0) and 1% aqueous solution of uranyl acetate, as previously described [37]. Samples were then dehydrated in alcohols with increasing concentration (from 50 to 100%) and embedded into methacrylate LR White as described previously [38]. Ultrathin sections (of 200–300 Å in thickness) were prepared using an LKB 3 ultratome and placed onto nickel grids with a formvar support. The ultrathin sections were stained with lead citrate using Reynolds' method [39] and analysed with a JEM-100B electron microscope.

### 3.7. RNA isolation

Four volumes of 5 M GTC solution were added to the mycobacterial liquid culture and mixed (5 M guanidine thiocyanate, 0.5% sodium N-lauroyl sarcosine, 25 mM sodium citrate, 1% Tween-80, 0.1 M β-mercaptoethanol). Cell suspensions were then centrifuged at 3000 r.p.m. for 30 min and the supernatant removed [40]. Total RNA was extracted from the pelleted bacteria disrupted in a BeadBeater (Biospec Products, USA) with 100 µm zirconium beads in Trizol (Invitrogen) followed by standard chloroform purification and isopropanol precipitation. Mycobacterial RNA was purified and DNase-treated using RNeasy columns (Qiagen), quantitated with the NanoDrop ND-1000 spectrophotometer (NanoDrop Technologies) and Agilent Bioanalyzer 2100. RNA samples were derived from NC bacilli with a high MPN/low CFU phenotype (CFU = 1.1 × 10^3^ and MPN = 7.0 × 10^6^) obtained after 41 days of incubation in potassium-limiting conditions, with resuscitation time intervals (Res 10, 24, 48, 96 and 192 h), together with log- and stationary-phase controls (±K^+^). RNA was also isolated from NC cells with a high MPN/high CFU phenotype (CFU = 7.0 × 10^6^ and MPN = 5.5 × 10^7^) derived from an independent model variant with aged inoculum (15 days preculture before inoculation to K^+^-deficient Sauton media). For all NC model variants, resuscitation time intervals and log/stationary-phase controls, RNA was extracted from three biological replicates from independent cultures. For the high MPN/low CFU NC model variant and resuscitation time intervals, 500 ng total RNA was amplified using the Bacterial MessageAmp modified Eberwine amplification procedure (Applied Biosystems) [41]. To dissect the NC transcriptional signatures from the high MPN/low CFU model variant, RNA from the corresponding log- and stationary-phase comparator cultures (±K^+^) were also amplified; all samples to be compared were extracted and amplified together.

### 3.8. Transcriptional profiling

Whole-genome microarrays generated by the Bacterial Microarray Group at St. George's (ArrayExpress accession number A-BUGS-23) were hybridized as described previously [42]. Briefly, 5 µg cDNA derived from each RNA sample and 2 µg *M. tuberculosis* H37Rv genomic DNA were labelled with Cy5-dCTP and Cy3-dCTP, respectively. RNA extracted from three biological replicates of all conditions were hybridized. Microarrays were scanned at 532 and 635 nm corresponding to Cy3 and Cy5 excitation maxima, respectively, using the Affymetrix 428 Array Scanner (MWG-Biotech). Comparative spot intensities from the images were calculated using Imagene 5.5 (BioDiscovery) and the data imported into GeneSpringGX v. 7.3.1 (Agilent Technologies). These data were normalized to the 50th percentile of all genes detected to be present and filtered to include genes flagged to be present in 80% of the arrays. Significantly differentially expressed genes were identified using ANOVA with Benjamini and Hochberg multiple testing correction (*p* < 0.05) and a minimum fold-change of 1.5 comparing profiles to log-phase controls or NC conditions.

To define the stresses conferred by potassium limitation, the transcriptional signatures of bacilli cultured in −K^+^ Sauton media in log phase (cultivated for 4 days) and in stationary phase (cultivated for 10 days) were compared to respective log-phase (4-day) and stationary-phase (10-day) cultures in +K^+^ standard Sauton media.

The hypergeometric function (hp) was used to determine the significance of overlapping gene lists, and transcriptional patterns were overlaid onto BioCyc metabolic maps using Pathway Tools network visualization [43]. Short time-series expression miner (STEM) [44] enabled significantly represented temporal expression profiles (*p* < 0.05 after Bonferroni multiple testing correction) to be recognized, identifying genes more than 1.5-fold differentially expressed at two or more time intervals after resuscitation. Significantly differentially expressed genes were hierarchically clustered using Cluster and the results displayed using Treeview [45]. Differentially expressed genes are detailed further in the electronic supplementary material, tables S1–S5. Fully annotated microarray data from this study have been deposited in BμG@Sbase (accession number: E-BUGS-151; http://bugs.sgul.ac.uk/E-BUGS-151) and ArrayExpress (accession number: E-BUGS-151).

### 3.9. Protein lysate preparation

Proteomics samples were extracted from NC state (high MPN/low CFU phenotype), log-phase (4 day) and stationary-phase (10 day) bacilli cultured in K^+^-limiting conditions, and from resuscitation time points Res 38 h, Res 63 h and Res 85 h. Two hundred millilitres of culture volumes at each time point were harvested in triplicate by centrifugation at 5000*g* for 5 min before washing twice with 20 ml PBS. The pellets were resuspended in 4 ml Tris-buffer sucrose (10 mM Tris, 250 mM sucrose, pH 7.0) and aliquoted into four 2-ml screw cap vials. Following centrifugation (5000*g*, 5 min) and removal of the supernatants, 200 µl 50% (v/v) suspension of 10 mM Tris, 250 mM sucrose, pH 7 and 100 µm zirconium beads were added to the bacterial pellets. The vials were bead-beated for 90 s at maximum speed followed by cooling on ice. Three hundred microlitres of lysis buffer (7 M urea, 2 M thiourea, 30 mM Tris, 4% CHAPS, pH 8.5) was added and mixed into each sample. The lysates were left on ice for 15 min before centrifuging at 10 000*g* for 5 min. The supernatants were collected and filtered through glass microfibre filters (Whatman). The lysate protein concentrations were determined by 2D-Quant (GE Healthcare). For western blot analysis, 10–20% Tris-glycine PAGE precast gels (Cambrex, Rockland, ME, USA) and the anti-Ald mouse monoclonal antibody HBT10 was used for detection and the development was performed by ECL (GE Healthcare).

### 3.10. Two-dimensional difference gel electrophoresis analysis

Samples were assayed in triplicate and included log phase, stationary phase, NC state (high MPN/low CFU phenotype) and three resuscitation time points: Res 38 h, Res 63 h and Res 85 h. Two-dimensional difference gel electrophoresis (DIGE) was performed as previously described [36]. Briefly, 50 µg lysates were prepared using the 2D Clean-up kit (GE Healthcare). Cy2, Cy3 and Cy5 minimal labelling was performed with 125 pmol of each CyDye according to the manufacturer's instructions (GE Healthcare). Isoelectric focusing with pH 4–7 IPG strips (GE Healthcare) was performed as previously described [46]. Cy2, Cy3 and Cy5 labelled samples were applied to the IPG strips during rehydration. The second dimension separation was performed in 10–20% SDS polyacrylamide gel electrophoresis gradient gels in the Protean IIxi system (Bio-Rad). Each two-dimensional gel included a Cy2-labelled internal standard (a pool of all experimental samples) to allow inter-gel variation to be corrected. Labelling was performed so that samples from each time point were labelled with both Cy3 and Cy5 to account for preferential labelling of proteins by either of the CyDyes. After electrophoresis, the gels were scanned with a Typhoon 9410 gel imager and the images were analysed using the Image Master Platinum v. 2.0 software (GE Healthcare). The Cy2-labelled standard was used for normalization and spots which displayed more than 1.5-fold difference in volume ratio compared with either log phase or NC state (one-way ANOVA *p* < 0.05) were selected for further characterization.

### 3.11. Protein identification by mass spectrometry

For protein identification by matrix-assisted laser desorption ionization–time of flight (MALDI-TOF) MS, silver stained [45] or Instant Blue-stained (Expedeon) spots were manually excised from the 2-dimensional electrophoresis gel and digested with trypsin as described previously [47]. Tryptic peptide mixtures were cleaned up using Proxeon Stage Tips and spotted on the MALDI target using α-cyanocinnamic acid as matrix. The peptide masses were analysed using an Ultraflex MALDI-TOF instrument (Bruker-Daltonics) and used to query the NCBI database (28 November 2009 release) by the Mascot search engine. Search parameters used were carbidomethylation of cysteine residues and allowing oxidation of methionine. Fragment mass error tolerance was 0.2 Da and up to one missed cleavage was allowed.

### 3.12. Glycerate kinase activity

The activity of glycerate kinase was determined using a luciferin–luciferase system measuring changes in ATP concentration regenerated during the conversion of phosphoglycerate to glycerate. *Mycobacterium tuberculosis* bacilli were disrupted in a BeadBeater (Biospec Products, USA) with 100 µm zirconium beads in dimethylsulfoxide. Cell debris and beads were removed by centrifugation at 3000*g* for 5 min. ADP 5 mM, 3-phosphоglycerate 1 mM and MgSO_4_ 1 mM were added to cellular extracts and ATP concentrations were measured after 60 min incubation at 37°C with a LUM-1 luminometer (Lumtek, Russia). Glycerate kinase activity was expressed in mol ATP regenerated per minute and enzyme activity was calculated per cell.

## 4. Results

### 4.1. Limitation of potassium induces *Mycobacterium tuberculosis* non-culturability on solid media in aerobic conditions

**Figure 1.**
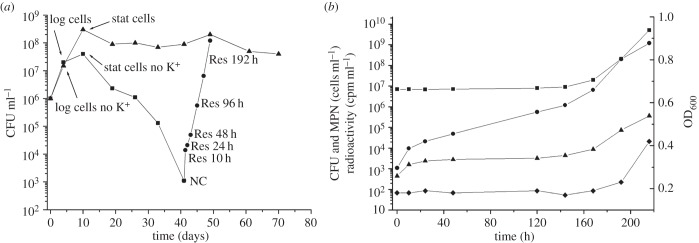
The formation and resuscitation of NC *M. tuberculosis* bacilli in potassium-limiting conditions. (*a*) Transition to an NC on solid media state in Sauton media lacking potassium (squares) and *M. tuberculosis* resuscitation after potassium re-introduction (circles) compared to mycobacterial growth in Sauton containing K^+^ (triangles). Measurements from a representative experiment in CFU ml^−1^ over 72 days of incubation. (*b*) Changes in cell characteristics during the transition from NC to actively growing state: most probable number (squares) (MPN ml^−1^); colony forming units (circles) (CFU ml^−1^); radioactive incorporation of uracil (triangles) (cpm ml^−1^); optical density (diamonds) (OD_600_). This experiment was repeated five times with similar results.

Replacing potassium ions with sodium ions in Sauton media resulted in similar *M. tuberculosis* growth kinetics between +K^+^ and −K^+^ cultures for 4 days (probably due to carry over of K^+^ with the inoculum); this period of log-phase growth was followed by a significant drop in growth rate in K^+^-deficient bacilli from 4 to 10 days after inoculation (figure 1*a*). After 10 days, *M. tuberculosis* CFU for K^+^-deficient media decreased further approaching a minimum at 39–41 days incubation (from 3 × 10^7^ CFU ml^−1^ in stationary phase to 1 × 10^3^ CFU ml^−1^ at 41 days), in contrast to Sauton medium containing K^+^ where bacterial numbers plateaued at 6 × 10^8^ CFU ml^−1^ after 41 days of incubation (figure 1*a*). This observation was replicated when bacilli were plated onto 7H10 and 7H11 solid media and Dubos-based agar. Bacterial CFU counts quickly recovered after washing and incubating bacilli in fresh liquid ‘resuscitation’ medium containing K^+^ (figure 1*a*). This resuscitation of growth occurred too quickly to be attributed to *M. tuberculosis* replication, suggesting that a large proportion of bacilli in potassium-limiting conditions although viable in liquid media were NC on solid media. We define non-culturability as a temporal inability of viable bacilli to form colonies on agar plates, which may be one of the key features of latent *M. tuberculosis* infection [48]. The existence of this NC population was confirmed by measuring the MPN of bacilli (in liquid media) to be 7 × 10^6^ ml^−1^ viable cells in comparison to 1.1 × 10^3^ CFU ml^−1^ (figure 1*b*): a high MPN/low CFU NC phenotype. This indicated that the vast majority of bacilli that were present after 41 days of incubation in the absence of potassium ions were NC on solid media. We found practically no difference between CFU counts (5 × 10^8^ CFU ml^−1^ on solid media) and MPN counts (7.2 × 10^8^ MPN ml^−1^ in liquid media) in bacilli cultivated in Sauton media containing potassium.

The resuscitation of this NC population (after transfer to K^+^-containing media) was characterized by further measuring optical density as a measure of bacterial growth and uracil incorporation to monitor transcriptional activity (figure 1*b*). The rate of radioactive uracil incorporation for NC cells obtained after 41 days of cultivation in K^+^-limiting conditions (CFU count 1.1 × 10^3^ ml^−1^; MPN count 7 × 10^6^ ml^−1^) was 2500 cpm ml^−1^, while the rate of incorporation for log-phase bacilli (CFU 8.2 × 10^6^ ml^−1^ MPN 1 × 10^7^ ml^−1^) was 175 000 cpm ml^−1^. Thus, this viable but NC sub-population that constitutes a large proportion of the total K^+^-limited cell population was at least 50 times less metabolically active than log-phase cells. In contrast to the fast recovery of bacterial CFU counts during resuscitation, neither optical density nor radioactive uracil incorporation nor MPN counts increased significantly until the eighth day (192 h) after transfer to K^+^-rich media (figure 1*b*). These indicators of growth demonstrated that after an 8-day period of resuscitation (where bacterial CFU counts alone increased), measurable metabolic activity returned as bacilli began to multiply.

### 4.2. Potassium limitation uniquely results in accumulation of a high proportion of non-culturable bacilli

To test our hypothesis that potassium levels may act as a specific stimulus for the generation of NC bacilli [32], we searched for a similar high MPN/low CFU phenotype after limiting other constituents of Sauton medium. We restricted key nutrients including nitrogen, carbon and phosphate sources but they resulted either in no significant CFU drop or led to a low MPN/low CFU phenotype, defined operationally here as irreversible cell death.

The importance of potassium limitation in this model of non-culturability was also confirmed by attempting to resuscitate bacilli in media lacking a source of potassium. Bacilli could not be resuscitated in the absence of K^+^; thus, the generation of NC bacilli in this *in vitro* model appears to be specific to the limitation of potassium ions.

### 4.3. Non-culturable cells are characterized by distinct cell morphology

**Figure 2.**
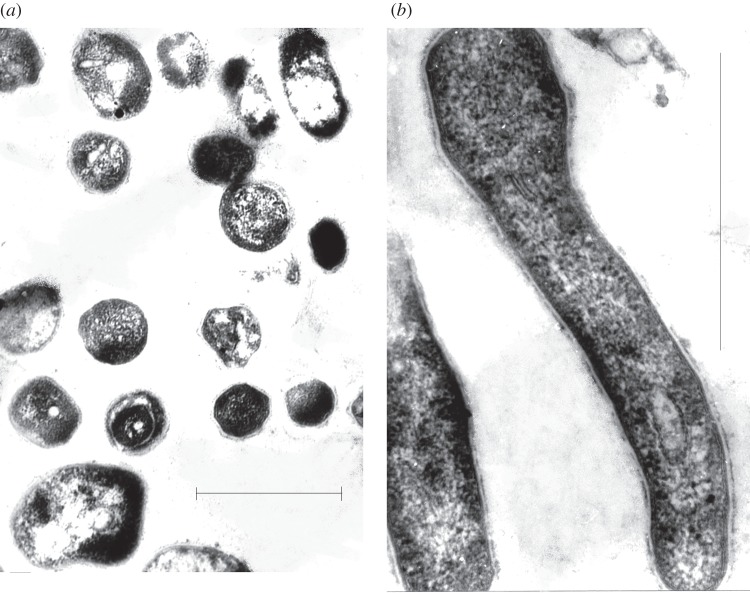
Changing mycobacterial morphology associated with non-culturablity. Transmission electron microscopy of (*a*) NC *M. tuberculosis* isolated from K^+^-deficient media and (*b*) early stationary-phase *M. tuberculosis* derived from K^+^-rich media. The scale bar corresponds to 1 µm.

Transmission electron microscopy revealed that the cell morphology of NC *M. tuberculosis* cells, derived from long-term incubation in the absence of potassium ions, was heterogeneous (figure 2*a*). The majority of NC bacilli appeared shorter and more spherical in comparison with bacilli grown in +K^+^ Sauton medium harvested in early stationary phase (figure 2*b*). The cytoplasm of NC cells was not homogeneous in contrast to early +K^+^ stationary-phase cells and contained vesicular electron transparent bodies. In some NC bacilli, the cytoplasmic membrane was observed to separate from the cell wall and was accompanied by loosening of the cytoplasmic membrane and ultrastructure organization of the cytoplasm. However, approximately 25–30% of bacilli possessed an intact cell wall and cytoplasmic membrane; these cells were characterized by a markedly condensed and compact cytoplasm compared to +K^+^ early stationary-phase bacilli.

### 4.4. Non-culturable cells are phenotypically tolerant to cell-wall-targeting antimicrobials

**Figure 3.**
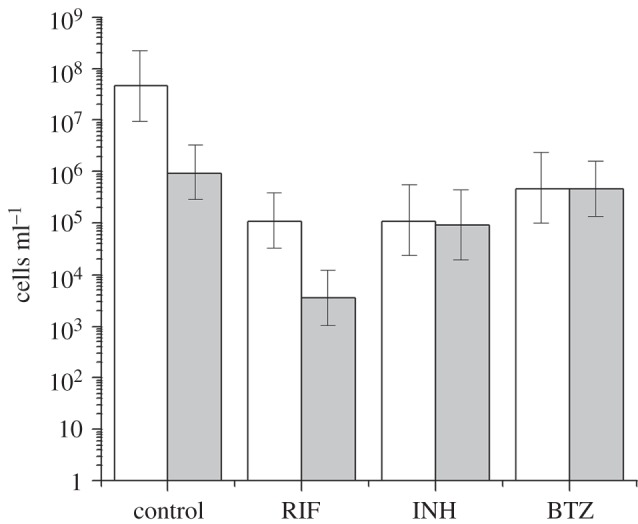
Antibiotic-mediated killing in actively growing and NC *M. tuberculosis.* Mycobacteria were exposed to 5 µg ml^−1^ RIF, 1 μg ml^−1^ INH or 0.2 μg ml^−1^ BTZ for 7 days. Viability was estimated by MPN assay and compared to drug-free controls. Open bars correspond to log-phase cells derived from K^+^-rich media, grey bars correspond to NC cells. The standard deviations of three independent experiments are marked.

We tested the activity of two first-line anti-tuberculosis drugs, isoniazid (INH) and rifampicin (RIF), and the newly developed compound benzothiazinone-043 (BTZ) [36] on NC and log-phase bacilli. The effectiveness of these antimicrobials was estimated by measuring the ability of NC cells to resuscitate after 7-day drug exposure by MPN assay. In contrast with plating on solid media, this approach enables the maximal number of potentially viable cells to be detected after antibiotic exposure. This allows non-culturability on solid media to be distinguished from drug killing, providing a suitable measure of the effect of these drugs on NC bacilli. A log-phase culture grown in +K^+^ Sauton medium at a similar cell density (estimated by MPN assay) was used as a reference. Seven-day exposure to RIF or INH caused approximately 2.5 log decrease in viable cells in log-phase bacilli compared with drug-free culture (figure 3). BTZ reduced the viable count of +K^+^ log-phase bacilli by two logs as measured by MPN. The same drugs were tested for their ability to kill NC cells derived from K^+^-deficient media. Both BTZ-043 and INH had little effect on NC cell viability: less than 1 log kill after 7-day exposure (figure 3). However, RIF efficacy remained similar to that observed for log-phase bacilli (approx. 2.5 log kill).

### 4.5. Transcriptome analysis revealed physiological adaptations of *Mycobacterium tuberculosis* bacilli to the non-culturable state

To characterize this model of dormancy further, transcriptome profiling was applied to define this NC state in comparison to log-phase bacilli. Total RNA samples were isolated from three biological replicates of cells representing the NC state after 41 days incubation in K^+^-deficient medium (high MPN/low CFU phenotype) and contrasted to log-phase bacilli cultivated in Sauton media containing potassium for 4 days (figure 1*a*); 830 genes were identified to be significantly induced and 864 genes repressed in NC bacilli (electronic supplementary material, table S1).

Unsurprisingly, transcriptional signatures previously identified to be involved in mycobacterial responses to conditions that slow growth were significantly enriched in the NC mRNA profile. Genes induced in models such as the enduring hypoxic response (hypergeometric probability (hp) 3.07 × 10^−26^) [49], nutrient starvation (hp 6.34 × 10^−20^) [10], the Wayne model (hp 4.74 × 10^−12^) [50] and stationary phase (hp 2.57 × 10^−10^) [51] overlapped significantly with genes induced in NC bacilli. Correspondingly, genes repressed during slow growth (hp 5.89 × 10^−24^) [52], nutrient starvation (hp 1.10 × 10^−24^) [10] or Wayne model NRP2 (hp 1.23 × 10^−38^) [50] significantly overlapped with those genes downregulated in NC bacilli.

To define the changing metabolic state of NC *M. tuberculosis*, we mapped the transcriptional adaptations characterizing NC bacilli to models of central carbon metabolism in *M. tuberculosis* [43,53,54] (electronic supplementary material, figure S1). Glycolysis and gluconeogenesis pathways were repressed in NC bacilli, with *pgi*, *fba*, *tpi*, *gap*, *pgk*, *pgmA*, *eno*, *pykA*, *aceE* and *lpdC* downregulated compared to log-phase bacteria; *pfkB* and *mez* were induced. Four genes implicated in the pentose phosphate shunt were also repressed (*fgd1*, *zwf2*, *tkt* and *tal*). The tricarboxylic acid cycle (TCA) was repressed (*citA*, *acn*, *icd1*, i*cd2*, *korA*, *korB*, *sucC*, *sucD*, *shdA*, *shdB*, *shdD*, *Rv0248c*, *fumC* and *gltA2* were downregulated in NC), as were *aceAa* and *glcB* of the glyoxylate shunt. Isocitrate lyase (*icl1*) was induced, corroborating that this alternative method of generating TCA intermediates is likely to be key to *M. tuberculosis* survival at multiple stages of disease [55]; *mutA* of the methylmalonyl pathway was also induced. A number of genes implicated in the catabolism of branched-chain keto and amino acids (*bkdA*, *bkdB*, *bkdC*, *fadE2*, *fadE13*, *accD2*) were upregulated. The induction of these genes (together with genes involved in the degradation of other amino acids) alongside the repression of fatty acid and mycolic acid biosynthetic pathways (*fabG1* and *inhA* from FASII, *fas*, *accD4*, *mmaA2*, *mmaa4*, *cmaA2*, *umaA*, *Rv2509*) suggests a switch to primary metabolites (including amino acids) as a carbon source in this NC state.

Processes linked to degradation and catabolism were also induced in dormancy. For instance, *pepD*, *pepR*, *htrA* and *clpC2*, encoding proteases and peptidases, were upregulated alongside *arcA* coding for arginine deiminase and *gcvB* encoding glycine dehydrogenase, which are involved in degradation of arginine and glycine, respectively. *hsaG* encoding an enzyme involved in the degradation of aromatic compounds was also induced.

The requirement for energy metabolism was reduced in this model of NC bacilli, mirroring other *in vitro* dormancy models. Genes classified into the functional categories [56] I.B energy metabolism (hp 7.19 × 10^−7^) and energy pathways GO : 0006091 (hp 8.49 × 10^−6^) were downregulated, as were genes encoding ATP synthase (*atpA*, *B*, *E*, *G*, *H*). Protein synthesis pathways were also repressed in NC bacilli: II.A.1 ribosomal protein synthesis and modification (hp 4.85 × 10^−11^), ribosome GO : 0005840 (hp 2.68 × 10^−10^), II.A synthesis and modification of macromolecules (hp 8.63 × 10^−5^). Rv2205c was induced in the NC state (although it did not pass the statistical testing threshold) and was also repressed on resuscitation from the NC state. Rv2205 has been recently annotated as a possible glycerate kinase (*glkX*) in *M. tuberculosis* [57] and may generate ATP from the transformation of phosphoglycerate to glycerate in non-replicating bacilli. We assayed this reaction biochemically comparing the activity of this enzyme in cytoplasm of NC bacteria compared to log-phase bacilli. For log cells, enzymatic activity of glycerate kinase (Rv2205c) was 1.67(±0.06) × 10^−20^ mol ATP cell^−1^ min^−1^, whereas for NC cells activity was 1.15(±0.01) × 10^−19^ mol ATP cell^−1^ min^−1^. The conversion of phosphoglycerate to glycerate was, therefore, seven times higher in NC than in log-phase bacilli, indicating that this pathway was activated in this model of *M. tuberculosis* dormancy.

Transcriptome analysis revealed that NC bacilli adopted an anaerobic respiratory state despite the presence of oxygen as NC bacilli were cultured in loose-capped flasks and shaken at 200 r.p.m. Genes encoding the proton-pumping NADH dehydrogenase I (*nuoA-N*) were significantly repressed, and the functional category of I.B.6.a aerobic respiration was significantly enriched in those genes downregulated in NC bacilli (hp 2.48 × 10^−10^), as was electron transporter activity GO : 0005489 hp (4.92 × 10^−5^). By contrast, the uncoupled non-proton-pumping NADH dehydrogenase II (encoded by *ndh*) was induced, highlighting this pathway as important for NC mycobacteria. In addition, genes encoding cytochrome *C* reductase (*qcrA*, *qcrC*), aa3 cytochrome *C* reductase (*ctaC*, *ctaE*) and cytochrome *bd* oxidase (*cydA*, *cydB*) were repressed, with *M. tuberculosis* probably switching to alternative electron acceptors. Genes coding for nitrate reductase (*narG*, *narH*) were significantly induced in NC bacilli. This transcriptional signature suggests that the limitation of K^+^ prevents bacilli from effectively using proton-motive force generated by respiration, driving bacilli to use NADH II and alternative electron acceptors.

Differential regulation of transcription factors accompanied these adaptations to the NC state. Genes coding for sigma factors A, B, E, F, G, H, I, L and M were induced (with *sigE*, *F*, *H*, *L*, *M* induced by more than fivefold and *sigB* induced more than 17-fold). Predicted regulons for SigG (hp 2.71 × 10^−8^) [58], SigH (hp 9.19 × 10^−7^) [59] and SigE (hp 5.98 × 10^−05^) [60] also significantly overlapped with genes upregulated in NC compared with log-phase bacilli. Conversely, *sigD*, *J* and *K* were repressed, alongside the predicted SigD regulon (hp 5.98 × 10^−6^) [61]. Genes encoding the MprA/B two-component regulatory system (*mprA*/*B*), which mediates a balance among stress responsive systems and is required for establishment and maintenance of infection in the host [62,63], were induced in NC. Other upregulated genes encoding two-component systems included *kdpD* (potassium transport) and *prrA* (required for successful macrophage infection) [64]. Genes encoding the transcriptional regulators KstR (transcriptional repressor controlling expression of cholesterol degradation pathways), RelA (key to the stringent response) and the redox-sensing regulator WhiB6 were also induced (*whiB1* was repressed in NC). Interestingly, only 11 genes of the DosR regulon, a set of 49 genes which are induced under hypoxia [51], were upregulated in NC cells.

Thus, the mRNA signature of NC *M. tuberculosis* in this model represents a unique combination of adaptations to the limitation of potassium, mediating the transition from log-phase bacilli (able to grow in liquid and solid media) to non-replicating NC bacilli that are unable to grow on solid media. Significantly, genes identified to be induced during macrophage infection (hp 6.78 × 10^−62^) [65] and (hp 3.67 × 10^−33^) [66] and in mycobacteria isolated from sputum (1.61 × 10^−18^) [31] were significantly enriched with genes upregulated in NC. Moreover, genes repressed intracellularly (hp 1.26 × 10^−95^ [65]; hp 6.57 × 10^−45^ [66]) and in sputum (hp 7.54 × 10^−66^) [31] were downregulated in NC. The large overlap between these transcriptional changes and mRNA signatures derived from infection models of *M. tuberculosis* disease suggests that similar mycobacterial phenotypes may exist during disease and may represent unrecognized ‘occult’ populations of NC cells *in vivo* [15,67].

### 4.6. Proteome analysis identified proteins with altered abundance in the non-culturable state

The expression profile of the NC state (high MPN/low CFU phenotype) was further defined by proteome analysis using the two-dimensional DIGE technique with samples collected from triplicate cultures. The abundant heat-shock proteins GroES, GroEL1, GroEL2 and DnaK were not included in the analysis as the labelling of corresponding spots was saturated by the scanning conditions applied. A total of 57 spots complied with the criteria for differential abundance. Of these, 39 spots were selected for identification and MS analysis led to positive identification of 32 spots; for the remaining seven spots, no significant hits were obtained. In two protein spots (Match IDs #1315 and #1344), two proteins (Ald, MoxR1 and AdhC, GuaB3, respectively) were confidently identified. The remaining 30 identified spots represented 25 unique proteins, reflecting that five proteins were identified in more than one spot. Despite the initial selection criteria, GroEL-1, GroEL-2 and DnaK were identified in three unsaturated spots, probably representing less abundant isoforms, and were not pursued further.

Proteome analysis demonstrated that seven proteins were increased in the NC state versus log phase: Ald, Wag31, RibA2, PpiA, FabG4, FixA and EchA6 (electronic supplementary material, table S2*a*). The increased abundance of Ald (Rv2780, l-alanine dehydrogenase) correlated with the transcriptome analysis and was verified by western blot analysis (electronic supplementary material, figure S2). Wag31 belongs to the DivIVA family of proteins known to regulate cell morphology in Gram-positive bacteria, which has been implicated in the mycobacterial response to oxidative stress [68]. RibA2 is predicted to be involved in the synthesis of riboflavin, a component in the cofactors FAD and FMN found in flavoproteins. PpiA is known to accelerate the folding of proteins. EchA6, FabG4 and FixA were all identified in two or more spots. Whereas the two EchA6 spots showed the same abundance pattern at different growth states, the individual FixA and FabG4 spots showed different profiles, probably reflecting that the isoform pattern of these proteins changes with different growth conditions. EchA6 and FabG4 are involved in fatty acid oxidation and fatty acid biosynthesis, respectively, and FixA is a probable electron transport flavoprotein.

A number of proteins decreased in abundance in the NC state compared with log phase (electronic supplementary material, table S2a): Adk, HspX, Rv2623, Gap, TB18.6, GcpE, KasB, GuaB1, Tsf, BfrB and FabG4. Adk (adenylate kinase) involved in nucleotide metabolism, the glycolytic enzyme Gap (glyceraldehyde 3-phosphate dehydrogenase) and the conserved hypothetical TB18.6 all correlated with the transcriptome dataset. Other proteins reflected the overall slowdown in metabolism at the NC state: GcpE involved in isoprenoid synthesis [69], KasB essential for fatty acid biosynthesis, GuaB1 with a role in GMP synthesis and Tsf involved in translational elongation all showed decreased abundance, as did BfrB (bacterioferritin) required for iron storage. HspX (identified in two spots) and Rv2623 are both predicted to be in the DosR regulon [70] and both proteins were less abundant in the NC state.

### 4.7. Defining the stresses imposed by limitation of potassium

To understand the metabolic changes which may lead to this NC state, we defined the stresses inferred from the *M. tuberculosis* transcriptional responses to potassium limitation in log phase and in stationary phase (figure 1*a*). In total 102 genes were induced and 80 genes repressed in log-phase bacilli in Sauton media without potassium ions relative to log-phase bacilli cultured in the presence of K^+^ (electronic supplementary material, table S3). Understandably, the most similar transcriptional response to the disruption of proton gradients by limiting K^+^ was low pH, with significant overlap between genes induced by low potassium and acid shock (hp 2.43 × 10^−13^) [71]. Other *M. tuberculosis* responses significantly enriched with genes induced by low K^+^ were nigericin (hp 4.10 × 10^−7^) and valinomycin exposure (hp 1.38 × 10^−5^) [72], both ionophore antibiotics that affect K^+^ transport. Therefore, potassium limitation in this model probably drives bacilli into an NC state by destroying *M. tuberculosis* membrane potential. This affects aerobic respiration pathways: genes coding for the proton-pumping NADH dehydrogenase I (*nuoAE-GN*) and cytochrome *C* reductase (*ctaE*) were repressed, and the functional category of I.B.6.a aerobic respiration was significantly enriched in those genes downregulated in log-phase −K^+^ bacilli (hp 1.48 × 10^−17^). Thus, *M. tuberculosis* probably switches to alternative electron acceptors: genes coding for ferredoxin (*fdxA*) and nitrite processing (*narK1*, *nirD*) were induced in log-phase bacilli lacking K^+^. The gene encoding the branched-chain keto acid dehydrogenase (*bkdA*) was also induced suggesting that alternative carbon sources were already beginning to be used in log-phase bacilli. As expected, the high-affinity ATP-driven potassium transport (Kdp) system (*kdpA–E*) was induced, reflecting bacterial adaptation to potassium limitation.

As incubation was extended and the absence of potassium ions advanced the mycobacterial physiology towards non-culturability, so the number of differentially expressed genes increased. Four hundred and two genes were significantly induced and 259 genes repressed in potassium-limited stationary-phase bacilli relative to +K^+^ stationary-phase bacilli (electronic supplementary material, table S3). As in log phase, the greatest overlap (with genes induced by low K^+^ in stationary phase) were signatures upregulated by treatments that impacted on the maintenance of membrane potential, such as the protonofore carbonyl cyanide m-chlorophenyl hydrazone (hp 1.95 × 10^−17^) [72] and low pH 4.8 (hp 4.42 × 10^−11^) [72]. Genes indicative of adaptations in metabolism such as *bkdA*/*B*/*C* (ketoacid catabolism) and *icl*/*glcB* (glyoxylate shunt) were induced, alongside induction of the non-proton-pumping type II NDH (*ndh*) and nitrate reductase (*narH*) and repression of type 1 NDH (*nuoD*/*E*/*I*/*L*) and cytochrome *bd* oxidase (*cydB*/*C*) encoding genes. Interestingly, 21 DosR-regulated genes were significantly downregulated in stationary-phase bacilli lacking potassium compared with +K^+^ stationary phase, with the DosR regulon and *M. tuberculosis* responses to low oxygen tension only induced in potassium-rich stationary-phase bacilli (hp 9.18 × 10^−14^ [73]; hp 2.59 × 10^−11^ [74]). From these observations, we hypothesize that the DosR regulon is only induced as oxygen tension decreases in aerobically respiring bacilli in +K^+^ stationary phase. By disrupting membrane potential, in this model by limiting K^+^ ions but also potentially in low-pH environments, bacilli switch to non-proton-pumping NADH dehydrogenase II and alternative electron acceptors where the induction of the DosR regulon is not required as bacilli enter stationary phase. Perhaps significantly, genes involved in protein synthesis were enriched in K^+^-deficient stationary-phase bacilli relative to K^+^-rich stationary-phase bacilli: translation factor activity GO : 0008135 (hp 1.25 × 10^−6^), ribosome GO : 0005840 (hp 6.71 × 10^−6^) and II.A.1 ribosomal protein synthesis (hp 8.74 × 10^−6^) [56]. This pattern was also mirrored by the SigD regulon, the expression of which often correlates with active growth (hp 1.44 × 10^−5^) [61]. Conversely, genes associated with slow growth were induced in +K^+^ stationary phase compared with −K^+^ stationary phase (hp 1.73 × 10^−6^) [52]. Thus, bacilli at this particular time interval (10 days after inoculation) appear to be more metabolically active in low potassium conditions than in standard Sauton media, highlighting differences in metabolic processes which lead to the establishment of two physiologically distinct stationary phases, in potassium-deficient and potassium-rich conditions.

### 4.8. *Mycobacterium tuberculosis* bacilli shift from dormant to replicating states on the reintroduction of potassium

This K^+^-limiting dormancy model provided the opportunity to study gene expression changes associated with transition from a slow/non-replicating NC state to re-growth in media containing K^+^. Accordingly, we defined resuscitation at five time points by transcriptome analysis, at 10, 24, 48, 96 and 192 h (figure 1*a*,*b*) after NC bacilli (high MPN/low CFU phenotype) were washed twice and inoculated into fresh ‘resuscitation media’ containing culture supernatant and Tween-80. No change in optical density or MPN was observed until 192 h (8 days) after resuscitation. However, growth on solid media (measuring CFU) increased by approximately 1 log in the first 10 h (too fast for multiplication) and continued to increase over 8 days from 1.1 × 10^3^ to 2.0 × 10^8^ CFU ml^−1^ (figure 1*b*). The incorporation of radioactively labelled uracil, as a measure of metabolic activity, increased marginally after 10 h of resuscitation, with no further increase until day 7. Thus, after transfer of K^+^-deficient NC bacilli to fresh ‘resuscitation medium’ (containing K^+^), the optical density, MPN and metabolic activity of bacilli did not start to increase for 7 days, however in that timeframe the CFU increased from 1.1 × 10^3^ CFU ml^−1^ to 6.5 × 10^6^ CFU ml^−1^; this resuscitation of bacilli from an NC state *in vitro* was unique to the limitation of K^+^. After 7 days of resuscitation, cell multiplication re-started and typical log-phase bacilli accumulated in the culture.

Spearman's rank correlation was used to enumerate the global changes in transcriptional profile between resuscitation time intervals relative to NC bacilli. The similarity in mRNA signature dropped from an average 0.961 (between NC biological replicates) to 0.855 (standard deviation of 0.017) at 96 h compared with NC. The correlation fell considerably to 0.569 (s.d. 0.007) at 192 h, indicating that the major changes in gene expression on resuscitation occurred between 96 and 192 h. This mirrors the pattern observed in optical density, MPN and cpm. Log-phase bacilli cultured in the presence of potassium were understandably the most dissimilar to NC bacilli (0.435, s.d. 0.018), with the correlation to NC rising as K^+^ was limited and incubation time increased as demonstrated by the log- and stationary-phase controls (table 1).

**Table 1.** The similarity in transcriptional signatures through non-culturability, resuscitation and active replication. Whole-genome Spearman's rank measurements comparing the average correlation of biological replicate transcriptional profiles from resuscitation (Res) and log/stationary-phase bacilli (±K^+^) with NC bacilli. Standard deviations are detailed.

similarity to NC	average correlation with NC	standard deviation
NC (between biological replicates)	0.961	0.030
Res 10 h	0.932	0.008
Res 24 h	0.932	0.006
Res 48 h	0.863	0.045
Res 96 h	0.855	0.017
Res 192 h	0.569	0.007
log-phase Sauton	0.435	0.018
log-phase −K^+^ Sauton	0.486	0.003
stationary-phase Sauton	0.530	0.008
stationary-phase −K^+^ Sauton	0.643	0.008

**Figure 4.**
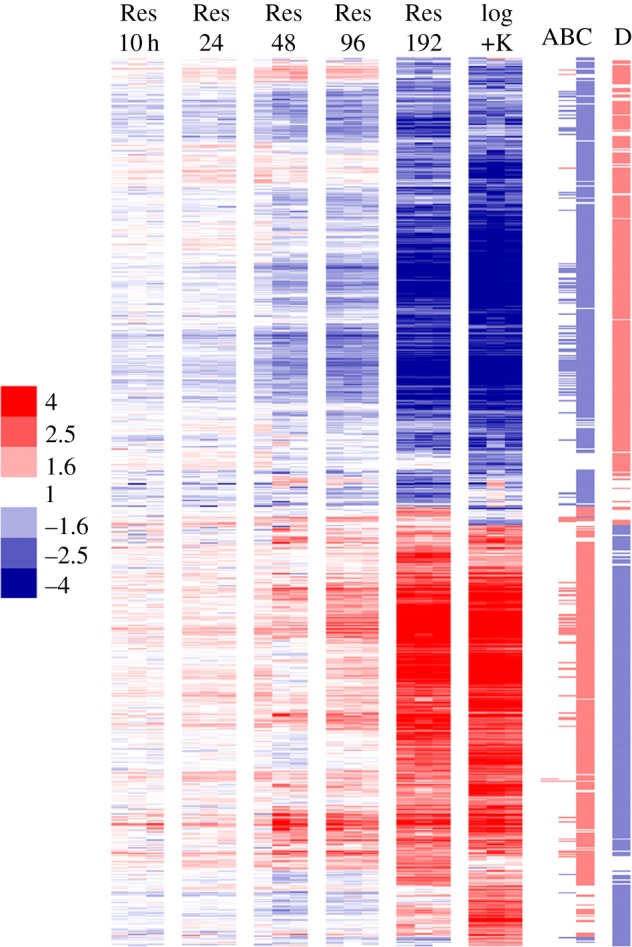
The transcriptional response of *M. tuberculosis* bacilli to resuscitation from an NC state. A total of 2146 genes significantly differentially expressed in NC bacilli compared with log-phase growth (log +K) or after resuscitation (Res) are shown. Biological replicates of conditions are displayed as columns, genes as rows. Red colouring represents induction of gene expression relative to NC, blue represents repression. Log2 expression ratios are plotted and the colour scale depicts fold change relative to NC. The columns on the right indicate in which comparison genes were identified to be significantly differentially expressed: (*a*) Res 24 versus NC; (*b*) Res 96 versus NC; (*c*) Res 192 versus NC; (*d*) NC versus +K^+^ log phase. The colouring of these markers indicates direction of expression (red, induction; blue repression).

**Figure 5.**
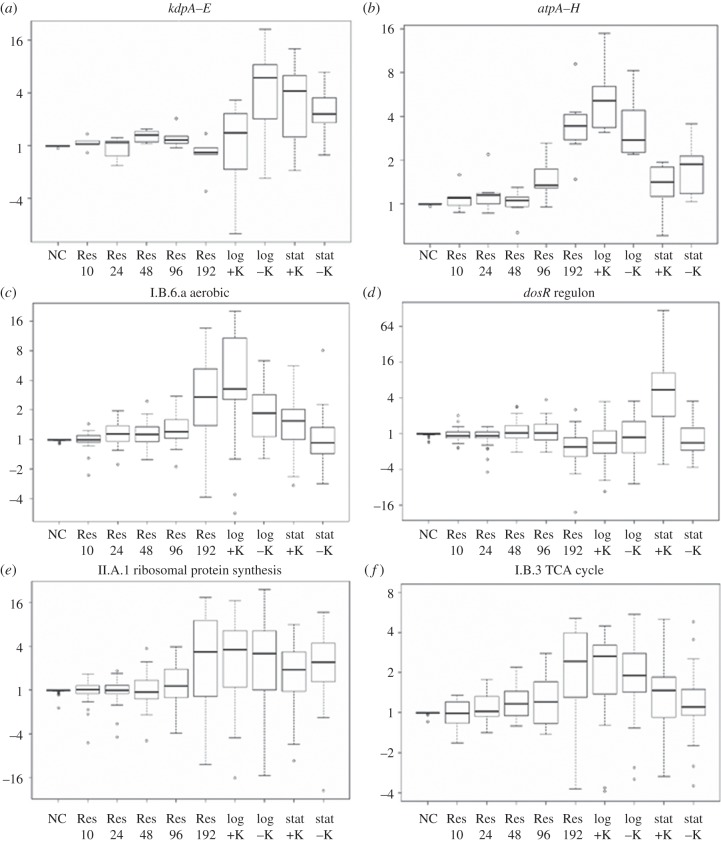
Dissection of the transcriptional adaptations to non-culturability and resuscitation. Box and whisker plots showing the distribution of expression ratios (log2 scale) of (*a*) *kdpA–E*; (*b*) *atpA–H*; (*c*) 21 aerobic respiration genes [56]; (*d*) *dosR* regulon [73]; (*e*) 45 ribosomal genes [56] and (*f*) 17 genes involved in the TCA cycle, in NC bacilli and after resuscitation (Res), together with log- and stationary-phase (stat) controls ± potassium. In all plots, the *y*-axis denotes fold change relative to NC, boxes encompass the 25th and 75th percentiles, whiskers have been set at 1.5× the range between these values and only outliers are shown as individual points.

Significantly differentially expressed genes were identified by comparing each time interval after resuscitation to NC state (figures 4 and 5). Two genes were significantly induced at Res 24 h time point, 68 upregulated at Res 96 h time point (108 repressed) and 769 genes induced at Res 192 h time point (881 repressed) (electronic supplementary material, table S4). Early responsive genes (within the first 96 h of resuscitation, before metabolic activity, MPN and optical density increased) included genes involved in the TCA cycle (*gltA2*, *icd2*, *sdhB*), FASII cycle (*hadA*, *inhA*), ATP synthesis (*atpE*) and NADH dehydrogenase I (*nuoD*, *nuoH*, *nuoM*), together with a small number of ribosomal genes (*rplI*, *rplL*, *rplM*, *rplP*, *rpsQ*). Of those genes repressed early on resuscitation were genes encoding NADH dehydrogenase II (*ndh*), ketoacid catabolism (*bkdA*), metal transporters (*ctpC*, *ctpH*, *ctpJ*) and the alternative sigma factors (*sigF*, *sigL*).

In contrast to early resuscitation stages (figure 4*a*,*b*), the transcriptional signature 8 days after resuscitation (Res 192 h) resembled log-phase growth (figure 4*c*,*d*) with genes significantly enriched in the functional categories of II.A.1 ribosomal protein synthesis and modification (hp 1.06 × 10^−11^) and 2 macromolecule metabolism (hp 1.49 × 10^−10^), and correlated to genes repressed in models of slowed growth such as nutrient starvation (hp 1.27 × 10^−27^) [10], slow growth (hp 2.00 × 10^−25^) [52], derived from sputum (hp 1.19 × 10^−49^) [31] or after macrophage infection (hp 4.39 × 10^−53^ [65]; hp 8.70 × 10^−33^ [66]). Genes involved in cell biosynthesis, lipid metabolism, translation and aerobic respiration were induced, thus this transcriptional pattern may be considered to detail pathways used by replicating bacilli. Indeed, transcriptome analysis revealed that 590 out of 769 genes found upregulated at Res 192 h time point were common with genes induced in log phase compared with NC. For example, genes reflecting the activation of main cellular metabolic processes such as Krebs cycle enzymes were activated during resuscitation. Contrastingly, the key enzyme of the glyoxylate pathway, isocytrate lyase (encoded by *icl*), was gradually repressed during resuscitation. Proton-pumping type I NADH dehydrogenase (encoded by *nuoA-M*) was progressively induced during resuscitation from Res 96 h to Res 192 h, indicating induction of a functioning aerobic respiratory chain. This was matched by the corresponding downregulation of components of the anaerobic respiratory chain (NADH dehydrogenase type II *ndh*, nitrate reductase *narGH*, cytochrome *bd* oxidase *cydC*), highlighting a reversal of the switch between aerobic and anaerobic respiration during the transition between NC and active growth. Genes encoding ATP synthase (*atpA-H*) were also upregulated for the synthesis of ATP by oxidative phosphorylation (figure 5). As expected, activation of transcription/translation and DNA replication pathways were also observed upon resuscitation. For example, 30S and 50S ribosomal proteins (figure 5), DNA polymerase III (*dnaN*) and replicative DNA helicase (*dnaB*), were induced, as were decaprenyl phosphoryl-β-d-ribose oxidase (*dprE1*) and decaprenyl phosphoryl-d-2-ketoerythropentose reductase (*dprE1*), targets for the new anti-tuberculosis drug BTZ, indicating a requirement for the synthesis of arabinogalactan to remodel or generate new cell wall components during replication. One out of five *rpf* genes, *rpfB*, was induced at Res 192 h alongside genes involved in phospholipid biosynthesis (*pssA* and *plsB2*), which may reflect the requirement for cell membrane remodelling during resuscitation.

To understand the order and timing of gene regulation as bacilli resuscitate, temporal profiles were identified that were significantly represented in the transcriptional dataset (electronic supplementary material, figure S3). Six profiles separated genes induced on resuscitation, differing by inflection point and magnitude. Interestingly, genes were induced in phases: for example, *icd1*, *icd2* and *sdhB* encoding part of the TCA cycle were induced at 10 h, increasing in magnitude over time. The genes coding for DNA polymerase A (*polA*) and nucleoside biosynthesis (*ndkA*) were induced after 24 h. Between 24 and 48 h, genes involved in lipid metabolism (*cmaA2*, *echA6*, *fadD31*, *fadE18*, *fas*), translation (*rplL*, *rplP*, *rplX*, *rpsA*, *rpsQ*, *trmU*) and sigma factor D (*sigD*) were upregulated. Genes encoding pathways for ATP synthesis, NADH dehydrogenase I, DNA replication and increased translational apparatus were induced between Res 48 h and 192 h, before restoration of a transcriptional signature resembling log-phase growth at Res 192 h. Strikingly, genes repressed on resuscitation (although differing by inflection point and magnitude) did not show a stepped downregulation over time, with genes repressed immediately after resuscitation decreasing steadily over time. These data suggest that bacilli begin to adapt transcriptionally to resuscitation at once with temporal changes only great enough to be detected by significance testing at later time intervals.

### 4.9. Resuscitation was associated with changes in protein abundance

Proteome analysis of *M. tuberculosis* resuscitation was performed in triplicate at three time points: Res 38 h, Res 63 h and Res 85 h. When comparing Res 85 h with NC state (high MPN/low CFU phenotype), 13 spots were found to be increased, including Gap, TB18.6, GcpE, KasB, GuaB1, Tsf, BfrB and one of the FabG4 isoforms, all of which were previously described to be decreased in the NC state compared with log-phase bacilli (electronic supplementary material, table S2*b*). In addition, PntAa, PurC, Rv3399 and one isoform of FixA were exclusively increased during resuscitation. PurC has a role in de novo purine biosynthesis; the increased abundance of this protein mirrored findings from the transcriptional profiling. PntAa (a subunit of the probable NAD(P) transhydrogenase) was induced in both protein and mRNA analyses; two other subunits PntAb and PntB were also found to be induced by transcriptomics. Rv3399, a probable S-adenosylmethionine-dependent methyltransferase involved in lipid biosynthesis, was increased, as were GuaB2, involved in GMP biosynthesis, and FixA, a β-subunit of an electron transfer flavoprotein.

Nine proteins showed decreased abundance when Res 85 h was compared with the NC state, including Ald, Wag31, RibA2, PpiA and spots representing EchA6, FabG4 and FixA isoforms. These proteins spots were also decreased in log phase compared with NC as previously described (electronic supplementary material, table S2*a*). Finally, the quinone reductase Qor and the adenylate kinase Adk were specifically decreased during resuscitation compared with NC bacilli.

### 4.10. Verification of this potassium limitation dormancy model

**Figure 6.**
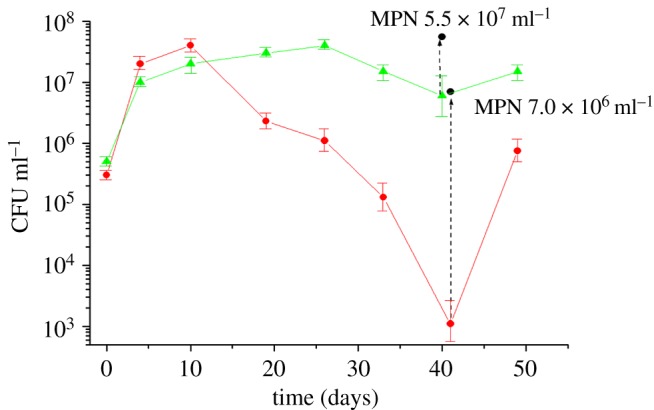
The definition of two model variations describing the generation and resuscitation of NC *M. tuberculosis* bacilli in potassium-limiting conditions. The high MPN/low CFU NC model variant (red circle) is plotted alongside an alternative high MPN/high CFU NC variant (green triangle). A 10-day-old inoculum was used to generate the high MPN/low CFU NC phenotype, whereas a 15-day-old inoculum resulted in a high MPN/high CFU NC phenotype. Measurements are detailed in CFU ml^−1^ over 50 days of incubation; corresponding MPN measurements for NC bacilli are marked. Experiments were repeated five times with similar results; a representative experiment is shown. Bars represent s.d. variation.

The number and MPN/CFU ratio of NC bacilli resulting from this K^+^-deficient model could be adjusted by altering the age of inoculum used to generate the NC phenotype (figure 6). We have described above NC bacilli with a high MPN/low CFU phenotype (7.0 × 10^6^ ml^−1^ and 1.1 × 10^3^ ml^−1^, respectively), where 99.7% of cells were NC. A second NC model variant (high MPN/high CFU phenotype) with a higher relative proportion of culturable bacilli (5.5 × 10^7^ MPN ml^−1^ and 7.0 × 10^6^ CFU ml^−1^, where 80% of cells were NC) was used to recover greater biomass from the K^+^-deficient NC model, thus enabling a comparator transcriptional dataset to be generated without the requirement for RNA amplification that was used to verify NC mRNA signatures. Transcriptional adaptations to the NC state (in comparison to log-phase bacilli) were broadly mirrored in both NC phenotypes, verifying the metabolic signatures associated with the NC state. Experiments to ensure the representation of RNA amplification and transcriptional analysis showed good correlation between model variants (hp 9.63 × 10^−71^; electronic supplementary material, table S5), thus validating the experimental approach to profile low numbers of NC bacilli. Furthermore, comparing transcriptome patterns between cultures with varying MPN/CFU ratio enabled mapping of the phenotypic state of NC bacilli without recourse to defining changes relative to log phase or over time.

To identify patterns of gene expression that may be specifically functionally relevant to non-culturability, genes were selected that were uniquely regulated in high MPN/low CFU versus high MPN/high CFU cultures. A number of transcriptional regulators of unknown function were induced in NC high MPN/low CFU (relative to the high MPN/high CFU phenotype), perhaps highlighting novel regulatory systems affecting adaptations to non-culturability (*Rv0067c*, *Rv0195*, *Rv0260c*, *Rv0302*, *Rv0377*, *Rv0465c*, *Rv1776c*, *Rv2282c*, *Rv2621c*, *Rv2640c*, *Rv3173c*). Genes encoding transcriptional repressors involved in redox regulation (*furA*) and lipid metabolism (*mce3R*) were also upregulated with increasing non-culturability. Strikingly, a large number of genes predicted to be involved in the transportation of moieties across the cell membrane were downregulated with greater ratio of NC bacilli (*arsB2*, *bacA*, *dppB*/*C*, *drrC*, *iniB*, *kdpC*/*F*, *nanT*, *oppC*, *pstC2*, *stp*), alongside multiple genes predicted to encode integral or transmembrane proteins (*Rv0544c*, *Rv0666*, *Rv0680c*, *Rv0987*, *Rv1216c*/*17c*, *Rv1226c*, *Rv1410c*, *Rv1517*, *Rv1610*, *Rv1616*, *Rv2219*, *Rv2326c*, *Rv2446c*, *Rv2571c*, *Rv2806*, *Rv3104c*, *Rv3524*, *Rv3645*, *Rv3689*). Thus, novel pathways affecting membrane permeability are likely to be important in the successful transition from logarithmically growing bacilli to NC states.

## 5. Discussion

The essence of the *in vitro* model developed in this study is the formation of *M. tuberculosis* sub-populations that are unable to grow on solid media, caused by the limitation of K^+^. We have identified potassium limitation as a specific environmental stimulus for the development of NC *M. tuberculosis* [32], extending our previous work with *M. smegmatis* [75] which mimics a remarkable feature of *M. tuberculosis in vivo.* Indeed, despite the fact that concentration of K^+^ in eukaryotic cells is probably sufficient for mycobacterial viability (*ca* 140 mM), mycobacteria captured by macrophages experience K^+^ deficiency in the phagosome [27,76] due to operation of a K^+^-efflux pump [27]. The combination of limitation of potassium in the extracellular medium and its acidification in the phagosome [27] may eventually lead to mycobacterial death or to development of dormancy. The validity of the K^+^-deficiency model is supported by a number of experimental findings. Thus, *kdpA-E* genes, encoding an inducible potassium ion transport system, that were upregulated in logarithmically replicating bacilli exposed to K^+^-deficient conditions were also induced in sputum [31] and in a murine hollow fibre model of disease [6], where the bacteria *in vivo* are extracellular.

Potassium ions are crucial for many cellular functions including the maintenance of intracellular pH and membrane potential [20–22]. Moreover, K^+^ is an essential ion for the function of the Na^+^K^+^ transporting ATPase. The inhibition of active transport of K^+^ into the cell is hypothesized to lead to membrane hyperpolarization [21], which in turn disrupts ion transport activity. This imbalance of K^+^-uptake in an *M. smegmatis trkA* mutant reduced the diffusion of large hydrophobic molecules and drugs such as rifampicin or novobiocin into the mycobacterial cell [21]. Additionally, there is evidence to suggest that decreased potassium levels inside a marine pseudomonad caused a reduction of amino acid uptake in bacteria [77]. Interestingly, mycobacterial phagosomes require K^+^ efflux from the bacterium for the successful generation of reactive oxygen species, and potassium deficiency results in increased survival of intracellular bacteria [78].

The highly tractable *in vitro* model of non-culturability developed in this study facilitates the interrogation of the physiological state of NC bacilli using both transcriptome and proteome analyses. The existence of sub-populations of non-growing bacilli have previously been reported, for example, in the Cornell and chronic murine models of infection [2,15] and in ‘occult’ bacterial subsets in sputa [67]. These sub-populations may also overlap with *M. tuberculosis* bacilli that persist through chemotherapy [79].

Potassium limitation resulted in significant changes in transcription during the early stages of *M. tuberculosis* adaptations to low K^+^ conditions (electronic supplementary material, table S3), as exemplified by induction of the high-affinity ATP-driven potassium transport (KDP) system (*kdpA-E*). Extended potassium limitation resulted in *M. tuberculosis* cell morphology changes and accumulation of NC bacilli round in shape, with intact membranes and condensed cytoplasm (figure 2). Similar coccoid cells with reversible culturability were isolated previously in *M. tuberculosis* cultures after prolonged storage in semi-anaerobic conditions [11] and as a result of lowering the pH [35]. Their appearance also resembled the coccoid-like forms isolated by Khomenko & Golyshevskaya [80] from organs of infected animals, which could be considered as dormant *M. tuberculosis* cells *in vivo*.

NC cells were tolerant to INH and BTZ, highlighting reduction of cell wall biosynthetic processes. At the same time NC cells remained sensitive to RIF, implying that some NC bacilli were still transcriptionally active but at a substantially reduced level (more than 50-fold reduction in ^3^H uracil uptake) or due to the presence of a residual sub-population of replicating cells in the NC culture. A similar drug tolerance pattern was observed in a starvation model [10] and 18b model of *M. tuberculosis* persistence [14]. Thus, NC cells derived from K^+^-deficient conditions were more able to survive cell-wall-targeting antibiotics in comparison with log-phase bacilli, thus displaying one of the predicted properties of persisting sub-populations *in vivo*, that of antibiotic tolerance. Despite fully aerobic conditions in this K^+^-deficient model, we observed a transcriptional profile that resembled *in vitro* systems modelling anaerobic conditions, in particular the Wayne model [50,51]. The NC metabolic state is characterized by a switch of C-metabolism to the glyoxylate shunt and downregulation of proton-pumping type I NADH dehydrogenase, electron transport chain enzymes and reduced ATP synthesis. Interestingly, this shift to anaerobic metabolism was also found for other models where dormant cell formation did not imply oxygen limitation [10,13,81,82]. This may also represent an *in vivo* metabolic state as similar gene expression changes were observed for intra-macrophage bacilli [66] and human sputum-derived bacilli [31]. Thus, such a ‘pseudo anaerobic’ response may be considered a general phenomenon manifested in lower activity of enzymes involved in aerobic metabolism and dependence on the anaerobic respiratory chain (including cytochrome *bd*, fumarate reductase, respiratory nitrate reductase and NADH dehydrogenase type II), which allows metabolic activity to be maintained at a low level in dormant states. We should note that K^+^-deficient Sauton medium does not contain any substrates that could be used as alternative electron acceptors. However, we cannot exclude that under transition to non-culturability, bacilli could produce their own metabolites that may be used as alternative substrates. Another possibility is that such acceptors originate from the human host (e.g. NO) and that induction of these pathways reflects the potential of NC cells for alternative respiration in the host.

Despite non-culturability on solid media, we detected ATP in NC cells but at reduced levels compared with log-phase bacteria. It is interesting to note that *mgtC*, coding for a membrane-bound protein that interacts with bacterial F_1_F_0_-ATP synthase, was upregulated in the NC state. This interaction results in the inhibition of the ATP synthase and a decrease of intracellular ATP levels [83]. This mechanism could be responsible for the reduction in ATP synthesis during the transition to this *M. tuberculosis* NC state. The question remains, how are NC cells able to generate ATP under repression of oxidative phosphorylation pathways? We suggest that transformation of phosphoglycerate to glycerate (by upregulating Rv2205c) identifies a possible alternative method of synthesizing ATP in NC bacilli.

The dramatic increase in CFU on resuscitation occurs soon after transfer of NC cells to +K^+^ liquid media supplemented by supernatant taken from actively growing cells. The nature of stimulating factors was not established but may involve resuscitation promoting factors (Rpf) proteins, as *rpf* knockout *M. tuberculosis* strains required wild-type supernatant for their resuscitation from an NC state [84]. Among the five Rpf (secreted proteins which stimulate resuscitation of dormant Actinobacteria) [85] homologues in *M. tuberculosis*, only *rpfB* was upregulated at 192 h of resuscitation. Although Rpfs are considered functionally redundant [86], individual Rpfs may be involved in cellular responses to particular stresses. In particular, *rpfA* was upregulated under anaerobiosis [50,73], and *rpfD* was induced after *M. tuberculosis* exposure to acidic pH [73]. *rpfB* and *rpfE* were upregulated during the adaptation of *M. tuberculosis* to the macrophage environment [66]. However, it is evident that *rpfB* upregulation was registered after 192 h of resuscitation, corresponding to active multiplication (electronic supplementary material, table S4). This parallels our previous findings on the involvement of Rpf proteins in the late phase of resuscitation in mycobacteria contributing to cell division processes [87]. Moreover, other low molecular weight factors (fatty acids and cAMP) might influence the early events of resuscitation [87].

In this work, we have studied a phenomenon of *M. tuberculosis* non-culturability on solid media *in vitro* which may be regarded as a model of latent TB infection. These *in vitro* NC cells resemble some of the assumed characteristics of *M. tuberculosis* bacilli responsible for latency: (i) bacilli possess a decreased ability to grow on standard solid media and require a special procedure of resuscitation for transition to active growth; (ii) these NC cells are defined by distinct cell morphology and tolerance to cell-wall-targeting drugs; and (iii) the NC state is characterized by downregulation of genes associated with central metabolic processes, however transcriptome analysis revealed genes activated in NC bacilli (or during transition to NC state), indicating their unique metabolic adaptation. We speculate that resuscitation represents a programmed modulation of metabolic processes that transition bacilli through challenging microenvironments during infection. Further understanding of these adaptations may provide novel strategies towards treating latent tuberculosis infection.

## Supplementary Material

Supplementary Figures

## Supplementary Material

Table S1

## Supplementary Material

Table S2

## Supplementary Material

Table S3

## Supplementary Material

Table S4

## Supplementary Material

Table S5
