# Supplementary material for: Potassium availability triggers Mycobacterium tuberculosis transition to, and resuscitation from, non-culturable (dormant) states
Source: Open Biol. 2014 Oct 15;4(10):140106. doi: 10.1098/rsob.140106 (PMC4221891; doi:10.1098/rsob.140106)
Supplement: Supplementary Figures [file rsob140106supp1.doc]

**Supplementary Figures**

**
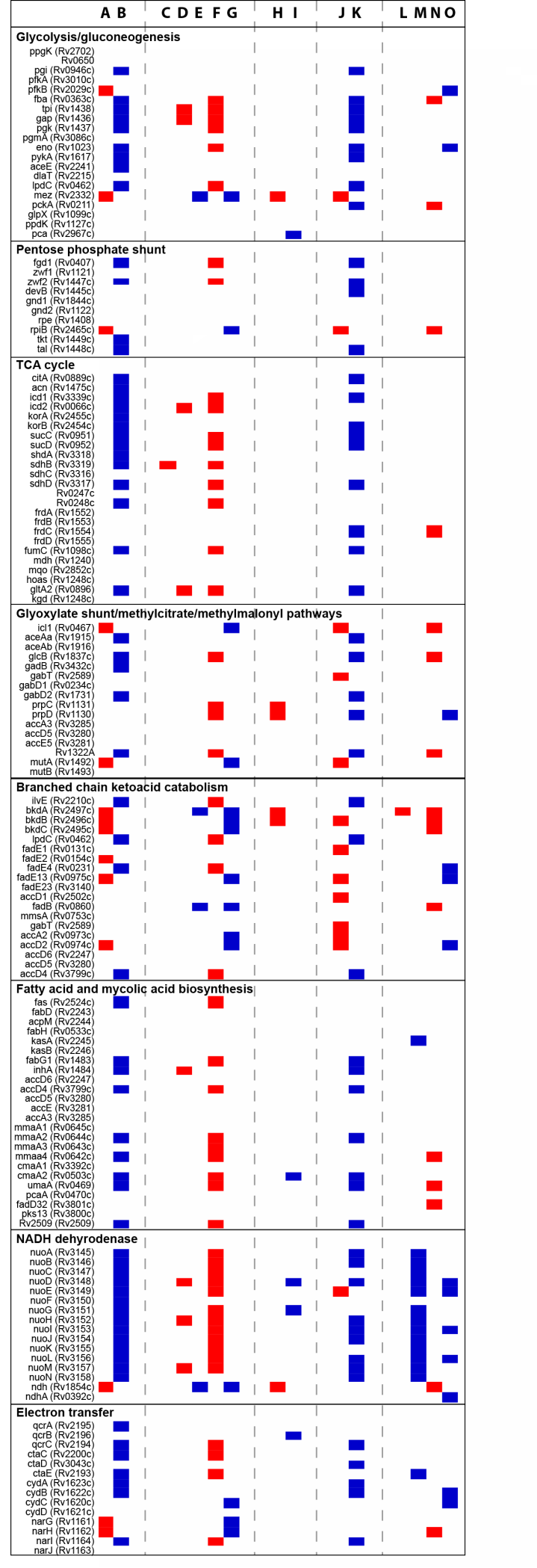
**

**Figure S1.** The adaptations to metabolic and respiratory pathways through non-culturability and resuscitation**.**

Genes significantly expressed in each comparison (A-O) are mapped to carbon and electron transfer networks [54,54]. Genes identified to be significantly induced are marked in red, repressed in blue. A/B: NC *versus* +K^+^ log phase bacilli; C: Res 24h *versus* NC; D/E: Res 96h *versus* NC; F/G: Res 192 *versus* NC; H/I: high MPN/high CFU NC *versus* +K^+^ log phase bacilli; J/K: high MPN/low CFU NC *versus* high MPN/high CFU NC; L/M: –K^+^ log phase bacilli *versus* +K^+^ log phase bacilli; N/O: –K^+^ stationary phase bacilli *versus* +K^+^ stationary phase bacilli.


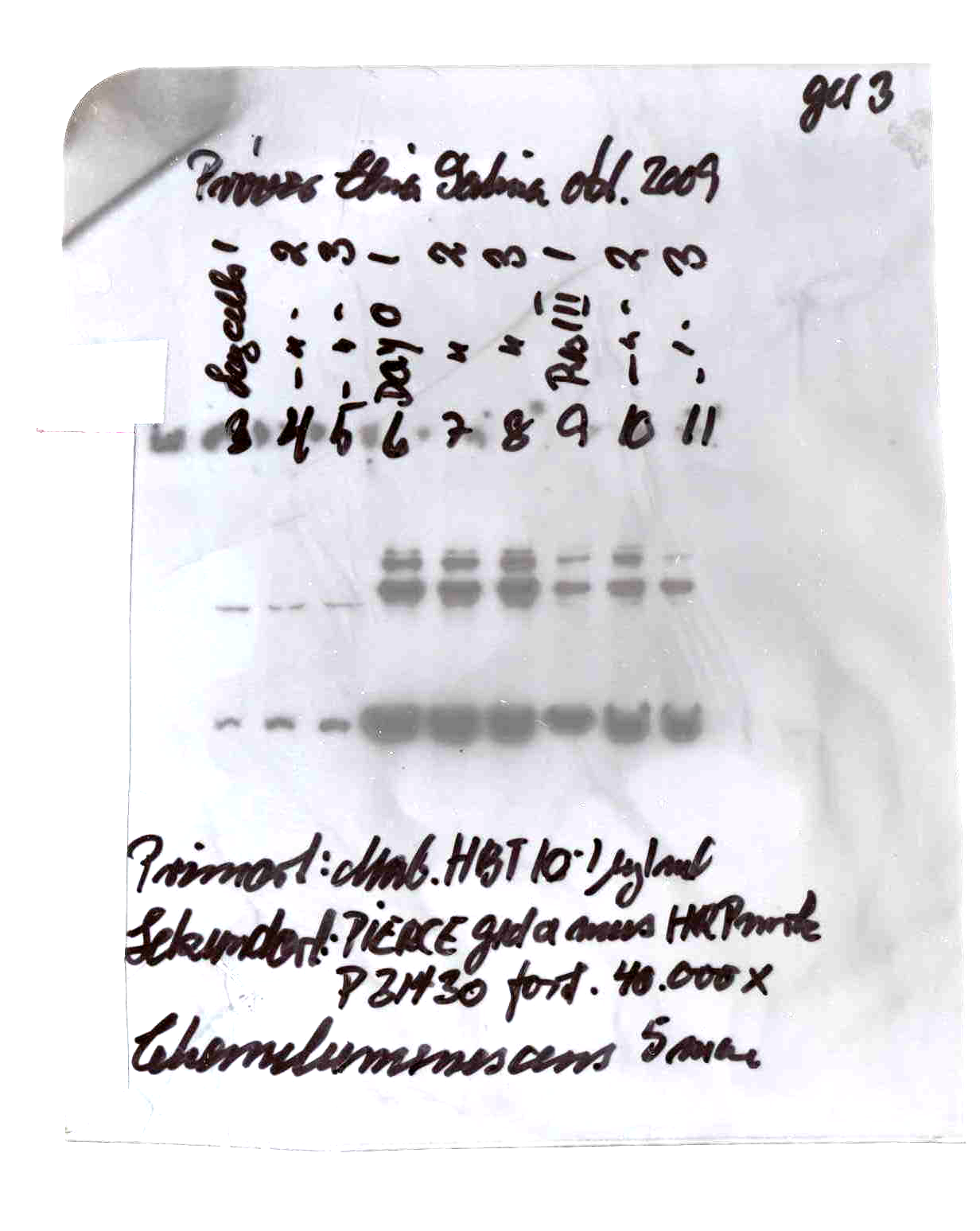


α-Ald

Log phase pse

NC state state

Res 85h 85h

**Figure S2.** Changing abundance of Ald estimated by Western blotting.

The presence of Ald in triplicate lysates from +K^+^ log phase (Log), NC state (NC) and resuscitation (Res 85h) cultures evaluated by Western blot using an anti-Ald mouse monoclonal antibody. Equal amounts of each sample (20 µg of total protein) were loaded.

**
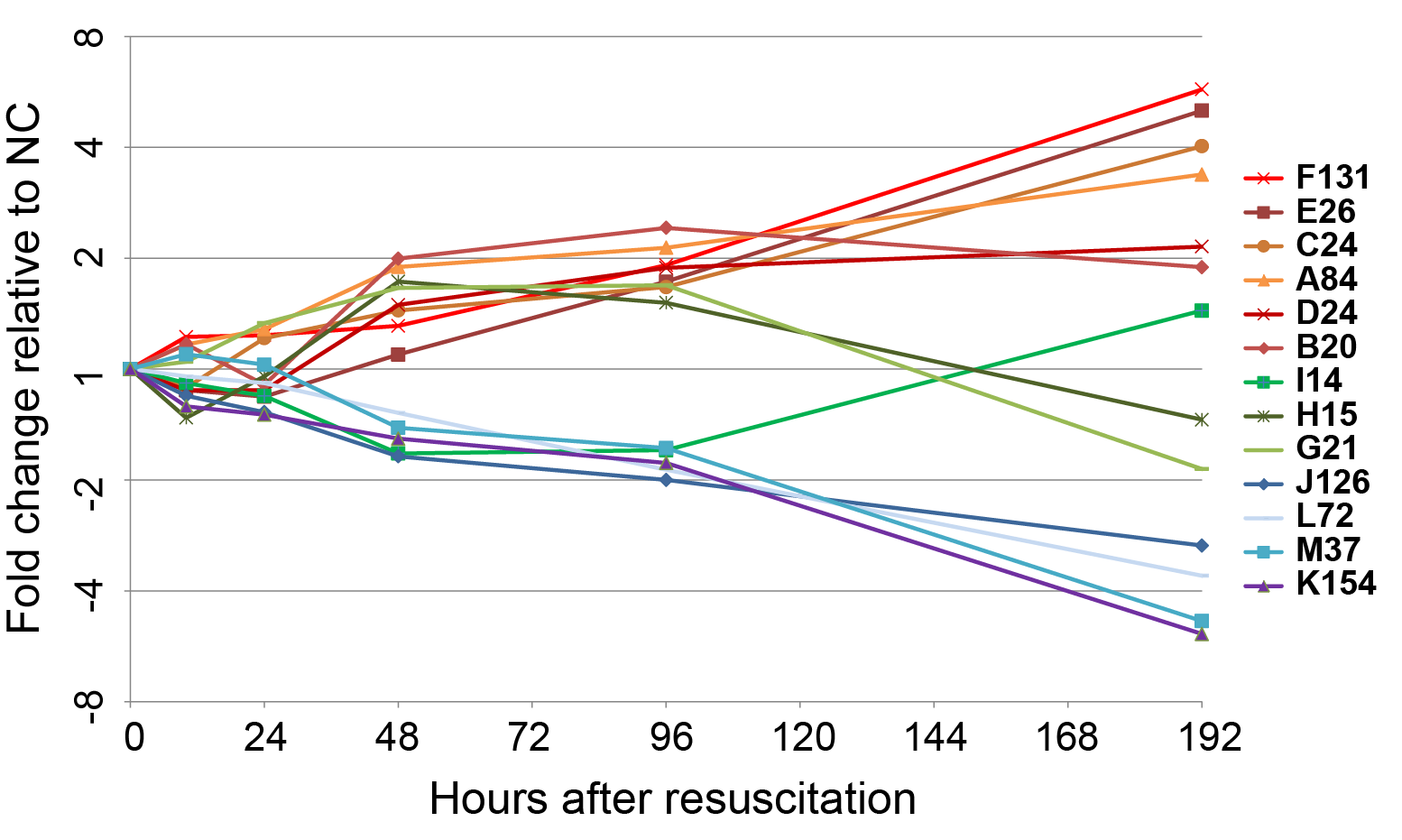
**

**Figure S3.** The temporal response to resuscitation.

Significantly defined gene expression profiles are plotted over time (hours after resuscitation). The thirteen profiles are labelled A-M followed by the number of genes assigned to each temporal pattern. The mean log2 expression ratio of genes within each profile are plotted as fold change relative to NC, and coloured by direction of expression after resuscitation (red colours highlight gene profiles induced with resuscitation; blue colours repressed with resuscitation; green colours denote gene profiles where the direction of expression changes over time).

**Supplementary tables**

**Table S1.** Differentially expressed genes in NC state compared to log phase growth in standard +K^+^ Sauton media.

**Table S2.** Proteins with changed abundance in the NC state and on resuscitation.

**Table S3.** Differentially expressed genes in +K^+^ compared to –K^+^ log and stationary phase cultures.

**Table S4.** Global changes in transcriptional profile during resuscitation.

**Table S5.** Differentially expressed genes from a high MPN/high CFU model variant compared to aerobic log phase and on resuscitation.
